# Effect of cleft palate type and manufacturing method on feeding plate adaptation: A volumetric micro‐computed tomography analysis

**DOI:** 10.1111/jopr.14027

**Published:** 2025-01-27

**Authors:** Osman Akıncı, Ece İrem Oğuz, Poyzan Bozkurt, Merve Berika Kadıoğlu, Mert Ocak, Kaan Orhan, Reha Şükrü Kişnişçi

**Affiliations:** ^1^ Graduate Oral and Maxillofacial Surgeon Antalya Turkey; ^2^ Department of Prosthodontics, Faculty of Dentistry Ankara University Ankara Turkey; ^3^ Oral and Maxillofacial Surgeon Istanbul Turkey; ^4^ Department of Orthodontics, Faculty of Dentistry Ankara University Ankara Turkey; ^5^ Department of Anatomy, Faculty of Dentistry Ankara University Ankara Turkey; ^6^ Department of Dentomaxillofacial Radiology Ankara University Ankara Turkey; ^7^ Department of Oral and Maxillofacial Surgery Lokman Hekim University Ankara Turkey

**Keywords:** 3D printing, adaptation, cleft lip and palate, feeding plate, micro‐CT

## Abstract

**Purpose:**

Feeding plates for cleft palate patients have been used by clinicians for many years to temporarily close the oro‐nasal communication until definitive treatment with surgical techniques. The current in vitro study aimed to evaluate the adaptation of the feeding plates manufactured by two different techniques for three cleft types.

**Materials and Methods:**

Feeding plates were manufactured with conventional compression molding (CM) and 3‐dimensional (3D) additive manufacturing on main models representing bilateral cleft, unilateral right, and unilateral left cleft types (*n* = 10). The 3D volumetric space between the feeding plate and the corresponding main model was measured by micro‐CT to evaluate the adaptation. The adaptation of the plates was assessed based on three different measurement regions: anterior, left, and right. Repeated measure analysis of variance (ANOVA), three factorial ANOVA, and post hoc Bonferroni tests were used as statistical analysis (*α* = 0.05).

**Results:**

CM groups showed higher volumetric space measurements between the base and master model than 3D groups regardless of measurement region and cleft type, which refers to misfit (*p* < 0.05). Cleft type differed in the adaptation of 3D groups yet not in CM groups (*p* < 0.05). The volumetric space evaluation for the right measurement region resulted in higher values regardless of manufacturing method and cleft type (*p* < 0.05).

**Conclusion:**

Considering that 3D‐printed feeding plates showed better adaptation compared to conventionally manufactured plates for all cleft types, 3D printing can be suggested as the manufacturing method of choice for feeding plates.

Cleft lip, cleft lip and palate (CLP), and cleft palate alone are non‐syndromic orofacial clefts that include a wide range of disorders affecting the lips and oral cavity.^1^ Although CLP are often seen together, their embryonic origin differs.^2^ A cleft lip is caused by the unsuccessful fusion of the maxillary and median nasal processes on one or both sides. On the other hand, a cleft palate is formed by the lateral palatal processes that do not fuse.^3^ The primary objective in treating an infant suffering from cleft palate is to ensure proper feeding.^4^, ^5^ A feeding plate is an appliance that is used to obturate the cleft and restore the separation between the oral and nasal cavities. This function is crucial for the adequate nutrition of the growing neonate since this prosthetic appliance creates a rigid platform toward which the baby can press the nipple and extract milk.^4^, ^6^

As with every prosthetic appliance, feeding plates require an intraoral impression. For CLP patients, this stage can be challenging with conventional impression techniques due to the risk of impression material aspiration.^7^, ^8^ In this regard, data acquisition by intraoral scanning is a significant advance for patients with CLP.^7^, ^9^ Parents and clinicians consider the impression of CLP children with intraoral scanning to be less invasive, less stressful, more comfortable, and quicker due to the reduced probability of repetition of the impression and decreased chair time.^9^, ^10^ After digital impression, the construction process of prosthetic appliances is enabled with computer‐aided design and computer‐aided manufacturing (CAD‐CAM) technology. The CAD‐CAM workflow includes a 3‐dimensional (3D) appliance design and computer‐aided production stage. Digital production techniques are divided into two main groups: subtractive (computerized numerical control milling) and additive (3D printing) manufacturing.^11^ In the subtractive technique, the prosthesis is milled from a pre‐polymerized acrylic resin block using milling machines, while in the additive technique, un‐polymerized light‐curable resin is 3D‐printed and polymerized by a light source after the digital manufacturing step.^12^

For all production techniques, polymethylmethacrylate (PMMA) is usually the material of choice for removable prosthetic appliances, including feeding plates. PMMA is readily available, has good strength, and can be fabricated with thin margins.^13^ On the other hand, a significant disadvantage of PMMA is the processing deformation, which may occur during the polymerization process of the acrylic, leading to dimensional changes and affecting the adaptation of the prosthesis.^14^, ^15^ Conventional and 3D printing PMMA processing techniques include the polymerization of acrylic via different heat and light sources in the production process, respectively.^14^ The post‐polymerization stage of PMMA in these techniques may cause different amounts of polymerization deformation.^16^, ^17^

Adequate retention is critical for feeding plates to maintain their function. Retention and adaptation of the plate are closely related since retention increases when the prosthetic appliance and the mucosa are in close contact and when the space between these is as small as possible to ensure the seal.^18^, ^19^ The mechanism behind this is related to the physical factors affecting the retention of a removable prosthetic appliance.^18^ The fluid film formed by saliva becomes thinner when the space between the base of the prosthetic appliance and the underlying mucosa decreases, increasing surface tension.^18^ The more significant the surface tension and the thinner the fluid film, the greater the retention.^20^ Therefore, the adaptation of the removable appliances, including feeding plates, is related to the space between the appliance and the underlying mucosa and can be evaluated by linear or 3D volumetric measuring of the space between them.^12^, ^14^, ^16^ Previous studies reported that adaptation of the prosthetic appliances may vary depending on the fabrication technique and the individual characteristics of the palate, such as the width, depth, and present undercuts.^14^, ^21^ Based on this information, the adaptation of feeding plates may vary depending on the cleft type. However, a study evaluating the adaptation of feeding plates in various cleft types and their relationship with different manufacturing methods needs to be included in the current literature.

Based on these considerations, the present study aimed to evaluate the adaptation of feeding plates manufactured by conventional and digital techniques. For this purpose, the adaptation of the feeding plate to the models with three different cleft types was evaluated by volumetric measurement using micro‐CT. The null hypothesis was that the adaptation of feeding plates would remain the same regardless of the cleft type and the manufacturing method.

## MATERIALS AND METHODS

The protocol of this study was approved by the Clinical Research Ethics Committee of Ankara University Faculty of Dentistry, Turkey (Protocol number: 36290600/43/2021). A plaster model previously obtained from a neonate with bilateral CLP by taking an intraoral C‐silicone impression (Zetaplus; Zhermack, ZetaPlus, Badia Polesine, Italy) was chosen for the present research. The plaster model was scanned with a desktop scanner (InEosX5; Sirona Dental Systems GmbH, Bensheim, Germany), and the standard tessellation language (STL) data was obtained. Then, the STL file was imported to Meshmixer (Autodesk Inc, San Rafael, CA, USA) software to optimize surface irregularities and reduce the undercuts to obtain the main model. For this purpose, the mesh quantity was reorganized, and the smooth tool (degree5) was used with a “shape preserving” option to protect the original model anatomy.

The model with bilateral CLP was duplicated, and the clefts were smoothed one by one to obtain two unilateral models. Thus, three main model designs with the same volume and outline form were obtained. Afterwards, main model designs were printed with a 3D stereolithography (SLA) printer (Formlabs Form 3; Formlabs Inc, Somerville, USA) using photopolymerizing gray resin (Grey Resin RS‐F2‐GPGR‐0, Formlabs Inc). After printing, the main models representing different cleft types were scanned with an intraoral scanner (TRIOS 4; 3shape, Copenhagen, Denmark), and the STL data were exported. Then, feeding plates in similar dimensions for each main model were designed using the Meshmixer software (Autodesk Inc).

The present study included two groups according to the feeding plate manufacturing method: 3D printing and conventional compression molding (CM). In the 3D group, the STL data of the feeding plates were exported to the 3D printer (Formlabs Form 3; Formlabs Inc) and feeding plates were printed with a 0.1 mm layer thickness using photopolymerizing resin (BioMed Clear Resin; Formlabs Inc.). After printing, the feeding plates were kept in 99% alcohol solution for 20 min (Form Wash; Formlabs Inc.) and subjected to UV light of 405 nm wavelength LED for 60 min (Form Cure; Formlabs Inc.).

In the CM group, the STL data of unilateral and bilateral feeding plates were transferred to a universal 5‐axis milling machine (inLab MC X5; Sirona Dental Systems GmbH, Bensheim, Germany) and milled from 30 wax blocks (CAD‐Wax; on dent, İzmir, Turkey) (*n* = 10). Then, milled wax feeding plates were converted to acrylic using the CM technique of heat‐polymerized PMMA resin (Akrodent, Koca Kimya ve Dental, Ankara, Turkey). The information regarding resin materials used is summarized in Table 1. The study groups and the abbreviations used are summarized in Table 2. A total of 60 feeding plates were produced with 10 plates per group.

**TABLE 1:** The properties and compositions of the resin materials used.

Resin type	Brand name and Manufacturer	Manufacturing method	Composition
Heat‐polymerized acrylic resin	Akrodent, Koca Kimya ve Dental	Conventional CM	Powder: Methyl methacrylate, ethyl hexyl acrylate, N‐octyl methacrylate Liquid: Methyl methacrylate, glycol dimethacrylate, dimethyl p‐toluidine
Photopolymerizing acrylic resin	BioMed Clear Resin; Formlabs Inc.	Stereolithographic 3D printing	50%–70% w/w Bisphenol A dimethacrylate, 7%–10% w/w 2‐hydroxyethyl methacrylate, 25%–45% Urethane dimethacrylate

Abbreviations: 3D, 3‐dimensional; CM, compression molding.

**TABLE 2:** The study groups of the study and their abbreviations.

	Cleft type		
Manufacturing method of feeding plates	Bilateral cleft (B) 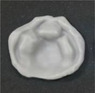	Unilateral left cleft (L) 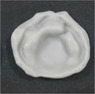	Unilateral right cleft (R) 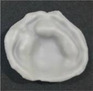
3D printing (3D)	3D‐B	3D‐L	3D‐R
CM	CM‐B	CM‐L	CM‐R

Abbreviation: CM, compression molding.

### Micro‐CT Scanning

**FIGURE 1:**
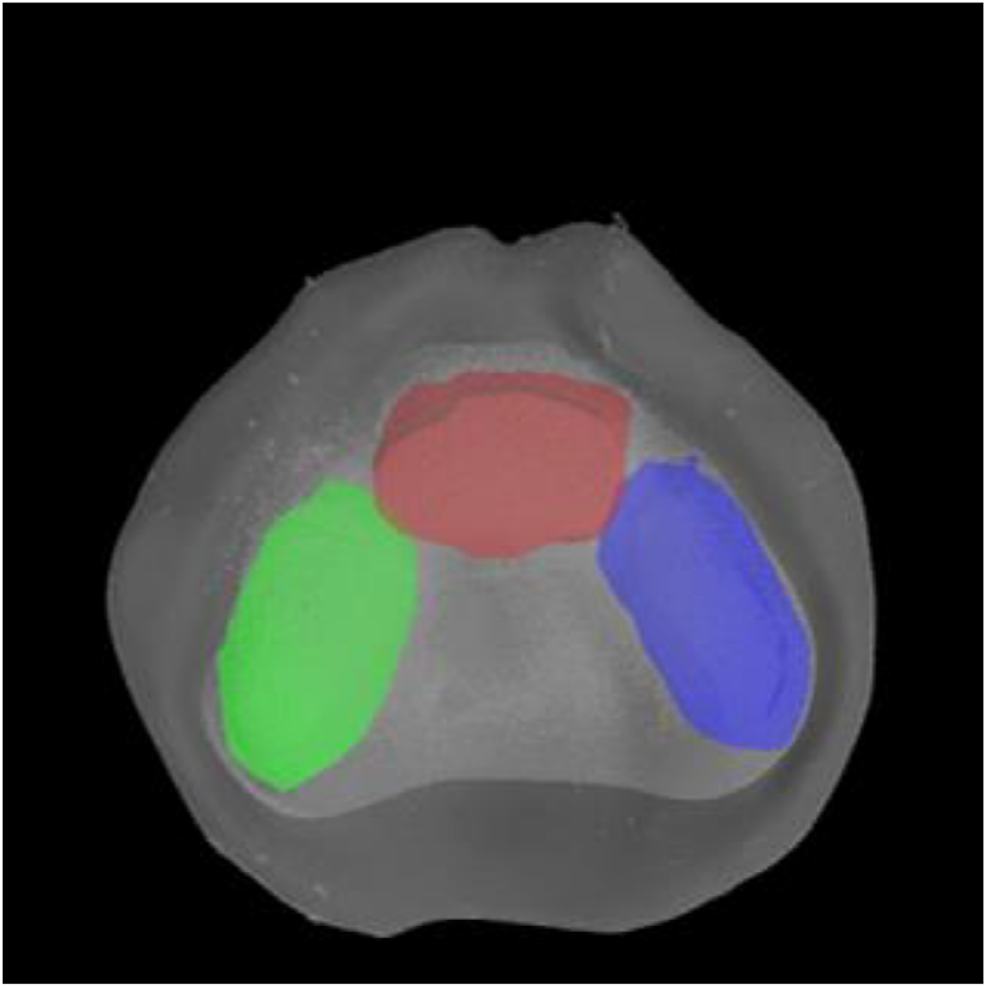
3D volumetric visualization of a representative scanned plate on the corresponding model. 3D, 3‐dimensional.

A high‐resolution desktop micro‐CT system (Bruker Skyscan 1275, Kontich, Belgium) was used to scan the feeding plates. The scanning conditions were as follows: 250 kV, 40 mA, 0.5 mm, no filter, 51 µm pixel size, and rotation at 0.2 steps (Figure 1). Each sample was rotated 360° within an integration time of 5 minutes, with about 1 hour mean scanning time. Before each scanning, the detector was air‐calibrated to minimize ring artifacts. Beam hardening correction and input of optimal contrast limits were utilized according to the manufacturer's instructions before each scanning and reconstruction of the samples. The main models were fixed onto the micro‐CT chamber with silicone adhesive to ensure the standard position of the models in all scans. It was crucial to perform the same region of interest (ROI) on each feeding plate and to calculate the volumetric space, accordingly. After the fixation, feeding plates were placed on the corresponding fixed model with finger pressure.

**FIGURE 2:**
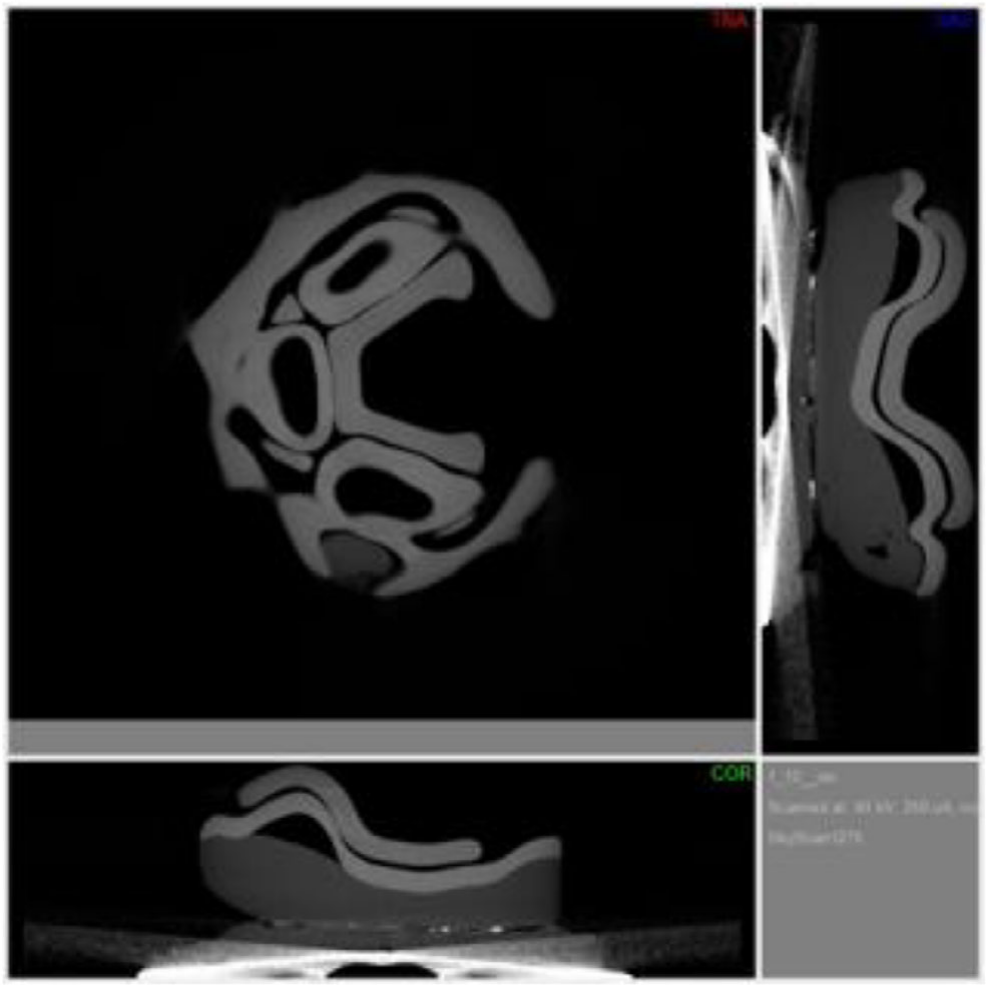
The ROIs of the anterior (red), left (green), and right (blue). ROIs, region of interests.

The visualization and quantitative measurements of the samples were carried out with the NRecon (ver. 1.7.1.0, SkyScan, Kontich, Belgium) and CTAn (ver. 1.19.11.0, SkyScan) software in which a modified algorithm described by Feldkamp et al.^22^ was used to obtain two‐dimensional (2D), axial, and 1000 × 1000‐pixel images. The ring artifact correction and smoothing reconstruction parameters were fixed at zero, while the beam artifact correction was set at 40%. The obtained images were reconstructed into axial slices with the NRecon software (Skyscan), followed by the CTAn (Skyscan) software, which was used for the 3D volumetric visualization and volumetric analysis of the space between the feeding plates and the main models. A suitable threshold was set to distinguish each feeding plate from its corresponding base scan. The reconstructed images were further processed in Skyscan CTVox for visualization. After reconstruction, three ROIs as anterior, left, and right were drawn using CTAn software to evaluate each sample's location‐based volumetric (mm^3^) adaptation (Figure 2). The same ROIs were used to ensure the standardized calculation on each sample. The original grayscale images of the main model and feeding plate in 3D volumes were processed with a Gaussian low‐pass filter for noise reduction with the global threshold method to calculate the space between them. An imposed image of black and white pixels only was obtained with this thresholding (binarization) process.

### Statistical analysis

Statistical analysis was performed by R v.3.5.3 (Microsoft Corporation, Redmond, WA, USA). The normality of the data was tested using Kolmogorov–Smirnov test. The effect of one within‐group variable (Measurement region three levels: anterior, left, and right) and two between‐group variables (Manufacturing method two levels: 3D printing and conventional resin; Cleft type three levels: bilateral, unilateral right, and unilateral left) on the adaptation of feeding plates were statistically analyzed by three‐factorial and repeated measures analysis of variance (ANOVA). Multiple comparisons were performed by the Bonferroni post‐hoc test (*α *= 0.05).

## RESULTS

The repeated measures ANOVA showed that interactions among the manufacturing method, cleft type, and measurement region variables were statistically significant (*p* < 0.05) (Table 3). Descriptive statistics are shown in Table 4, which compares the plates' adaptation regarding the manufacturing method, cleft type, and measurement region. Higher mean volumetric space measurement values between the plates and models presented in this table indicate increased misfit and decreased adaptation.

**TABLE 3:** The results of repeated measures ANOVA.

Effect	Sum of squares	Degree of freedom	Mean sum of squares	F ratio	*p* value
Manufacturing method	7.7470	1	7.7470	643.21	0.0001
Cleft type	0.2520	2	0.1260	10.46	0.0001
Manufacturing method *Cleft type	0.0989	2	0.0494	4.11	0.022
Error	0.6504	54	0.0120		
Measurement region	5.0670	2	2.5335	310.44	0.0001
Measurement region *Manufacturing method	0.0427	2	0.0214	2.62	0.078
Measurement region *Cleft type	0.1021	4	0.0255	3.13	0.018
Manufacturing method * Cleft type* Measurement region	0.1618	4	0.0405	4.96	0.001
Error	0.8814	108	0.0082		

Abbreviation: ANOVA, analysis of variance.

**TABLE 4:** Mean and ±SD for gap measurements (mm^3^).

Measurement region			
Groups	Anterior	Left	Right
	Mean ± SD	Mean ± SD	Mean ± SD
3D‐B	1.4 ± 0.1**^Aab^**	1.4 ± 0.1**^Aa^**	1.9 ± 0.1**^Ba^**
3D‐L	1.3 ± 0.04**^Ab^**	1.3 ± 0.04**^Aa^**	1.7 ± 0.2**^Bb^**
3D‐R	1.5 ± 0.07**^Ba^**	1.4 ± 0.04**^Aa^**	1.7 ± 0.1**^Cb^**

CM‐B	1.8 ± 0.09**^Aa^**	1.9 ± 0.06**^Aa^**	2.1 ± 0.1**^Ba^**
CM‐L	1.8 ± 0.09**^Aa^**	1.8 ± 0.09**^Aa^**	2.1 ± 0.1**^Ba^**
CM‐R	1.8 ± 0.09**^Aa^**	1.8 ± 0.06**^Aa^**	2.1 ± 0.1**^Ba^**

*Note*: Different superscript uppercase letters (A, B, C) in the same line and different superscript lowercase letters (a, b) in the same column indicate statistically significant difference (*p *< 0.05).

Abbreviations: 3D‐B, 3D printing‐bilateral cleft; 3D‐L, 3D printing‐unilateral left cleft; 3D‐R, 3D printing‐unilateral right cleft; CM‐B, compression molding‐bilateral cleft; CR‐L, compression molding‐unilateral left cleft; CR‐R, compression molding‐unilateral right cleft; SD, standard deviation.

When comparing measurement regions (within‐group variable), the highest mean volumetric space measurement was seen in the right measurement region in all groups, irrespective of the cleft type and manufacturing method. There was no statistical difference between anterior and left measurement regions for all groups (*p* > 0.05) except 3D‐R, for which the left measurement region resulted in the lowest mean space (*p* < 0.05).

When the cleft types are compared for feeding plates manufactured with 3D printing, the mean volumetric space measurement of the 3D‐B group was comparable with the 3D‐L and 3D‐R groups in the anterior measurement region (*p* > 0.05) (Table 4). However, the 3D‐R group showed a higher mean volumetric space than 3D‐L for this region (*p* < 0.05). In the left measurement region, all 3D‐printed feeding plates showed similar results regardless of the cleft type (*p* > 0.05). In the right measurement region, the 3D‐B group showed higher mean results than the other two groups, which were not statistically different (*p* > 0.05). On the other hand, differences between mean volumetric space measurements for different cleft types were insignificant for conventionally manufactured feeding plates in each measurement region (*p* > 0.05) (Table 4).

**FIGURE 3:**
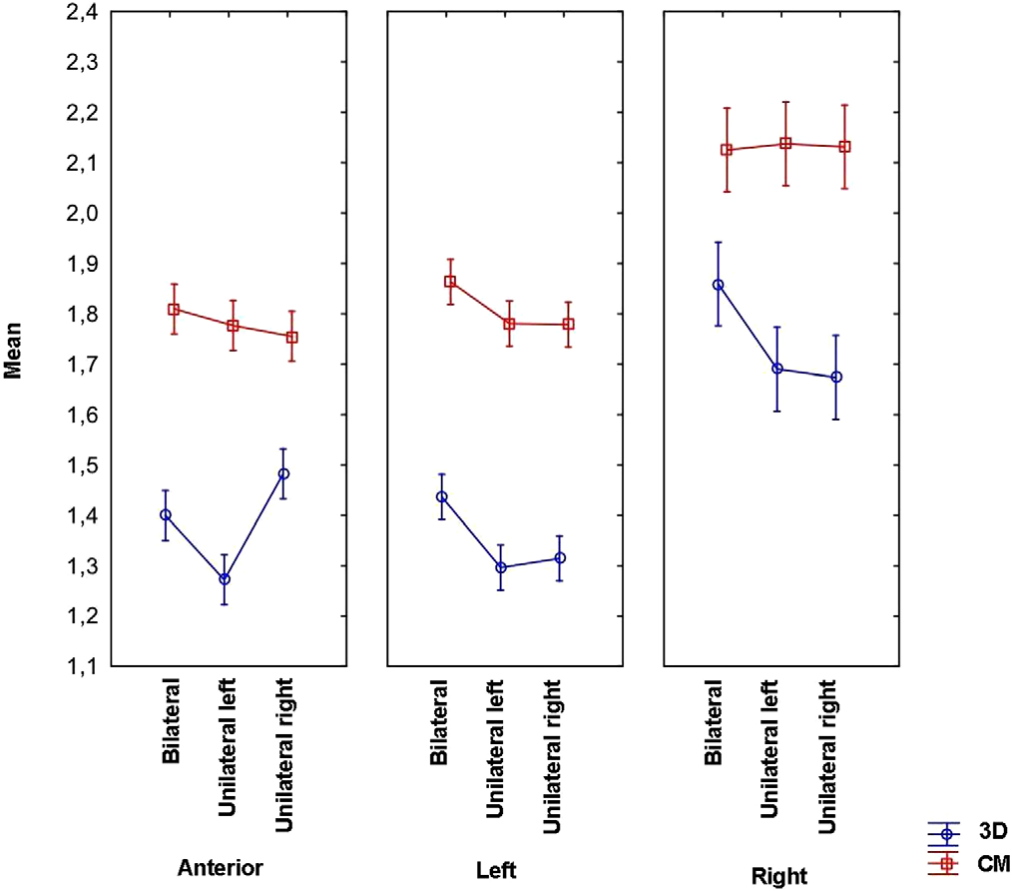
The comparison of 3D printing and CM groups regarding different measurement regions (anterior, left, and right) and cleft types (bilateral, unilateral left, unilateral right). 3D, 3‐dimensional; CM, compression molding.

The manufacturing method comparison revealed that plates obtained with 3D printing showed significantly lower volumetric space between the base and model than those obtained with conventional methods in all cleft types and measurement regions, meaning a better adaptation (*p* < 0.05) (Figure 3).

## DISCUSSION

Individual feeding plates are used to overcome the feeding problems prior to comprehensive surgical treatment of babies born with CLP.^6^, ^23^, ^24^ The seal of these plates is important for the adaptation, which is in direct relationship with the space between the mucosa‐cleft area and the plate. The present study was carried out to evaluate the adaptation of feeding plates manufactured for three different cleft types with two different manufacturing methods. In vitro micro‐CT scanning and volumetric measurement methods were used to calculate the 3D space between the plate and the model representing the mucosa. The adaptation of the feeding plates differed according to cleft type, manufacturing method, and measurement region. Thus, the null hypothesis was rejected.

Feeding plates are prosthetic appliances with features similar to complete dentures used in adult edentulous patients. The main difference between these two is their function, which is the obturation of the cleft area for feeding plates.^25^ Since the obturation of the base is related to the adaptation, basic principles for conventional dentures are valid for the current study.

CAD‐CAM dentures have recently been introduced in removable prosthodontics and investigated frequently. Although some authors stated no difference between CAD‐CAM dentures and conventional dentures,^26^ several studies underlined the increased adaptation with CAD‐CAM dentures.^16^, ^27^, ^28^, ^29^ Our results that represent a higher adaptation for 3D printed feeding plates compared to conventional ones are in line with these previous findings. A recent study by Aretxabaleta et al.^30^ investigated the accuracy of feeding plates manufactured by SLA, direct light processing (DLP), subtractive manufacturing (SM), and conventional manufacturing by cold polymerizing PMMA. They stated that DLP and SM techniques showed superior trueness than feeding plates manufactured by the conventional method and SLA. They stated that SLA demonstrated the lowest trueness. These findings conflict with our results, which showed higher adaptation for SLA compared to the conventional method. A possible explanation for this conflict may be the difference in manufacturing processes these two studies employed. As such, in the study by Aretxabaleta et al.,^30^ conventional plates were produced by an experienced dental technician. The standardization of the results may not be ensured as each plate may result in a different thickness, which affects the polymerization shrinkage and adaptation.^16^, ^21^ On the other hand, in our study, conventional plates were standardized by using wax plates obtained by SM of the same STL data. The main models were also standardized by 3D printing of each cleft‐type group followed by optical scanning of the cluster model. According to our findings, optical scanning followed by 3D printing can be recommended as the manufacturing method of choice for feeding plates, considering the higher adaptation results. The superiority of the adaptation in 3D groups can be associated with the precision of the surface processing capacity of additive manufacturing.^31^

The adaptation of bases may vary in different palatal regions.^14^, ^21^, ^26^ The present study evaluated the adaptation of plates in three different regions as anterior, left, and right. Although 3D‐printed plates showed better adaptation than conventional ones, all tested groups were less compatible in the right region of the plate. An undercut in the right posterior vestibular sulcus region may be the reason for this finding. Palatal depth and type were also reported to affect the adaptation of dentures.^21^ Based on this information, the adaptation of three different cleft types was investigated in the present study. 3D‐printed plates showed discrepancies for different cleft types in the anterior and right regions, while conventionally manufactured plates showed similar adaptations to all cleft types. According to this finding, it can be said that the adaptation of 3D‐printed plates was affected by cleft type, unlike conventionally manufactured plates. The same STL data was used to manufacture the plates for both techniques. However, post‐productional problems related to the manufacturing method, such as polymerization shrinkage, occur after both techniques.^16^ Polymerization shrinkage was previously reported to affect dimensional stability and adaptation of the bases.^16^, ^26^ The differences in regional adaptation between 3D printing and conventional methods may have derived from the differences in polymerization shrinkage processes. Also, polymerization shrinkage of 3D‐printed bases may have been affected by biomaterials used in the fabrication and post‐curing processes performed after production.^14^, ^32^ Yet, 3D‐printed resin and post‐curing process were not diversified in this study. Future studies may focus on the effect of these variables on the adaptation of feeding plates.

Previous studies employed conventional analog or 2D digital methods to evaluate base adaptation based on linear measurement of the vertical distance between the model and base.^14^, ^33^ However, the volumetric space that determines the adaptation occupies a 3D space, and linear measurements may be insufficient and misleading when evaluating the adaptation.^14^ In the current study, the gap was evaluated using a volumetric 3D measurement with micro‐CT. The innovative method used in this study may lead to new approaches when evaluating the adaptation of feeding plates.

Standardization is substantial in ensuring clinical validity and consistency of the results in in vitro experiments. A single model with a bilateral cleft was copied by flattening one cleft at a time to produce two unilateral cleft models. Thus, models with similar volume and surface topography were obtained to ensure standardization. Also, all plates were manufactured from the same STL and were evaluated using the same model for adaptation in each group. However, as in all in vitro studies this study provides limited in vivo insights due to the lack of intraoral conditions such as saliva, mucosal resilience, and the pressure exposed during sucking movement in feeding plates.  Thus, how the results will relate to in vivo conditions remains unclear. Further research incorporating in vivo conditions is recommended to validate the findings of the present study. Also, the present study used models with three different cleft types to evaluate the adaptation of feeding plates. Yet, CLP may be present in many clinical and subclinical phenotypes.^34^ The width and boundaries of the cleft may influence the adaptation. Future studies may focus on the effect of these variables on the adaptation of feeding plates manufactured with different techniques.

The current literature lacks a reference gap value for evaluating the adaptation of removable appliances. Although determining a clinically acceptable volumetric space value between the base and mucosa based on the present in vitro results was not feasible due to the absence of clinical evidence, this study may serve as a foundation for subsequent in vivo research.

## CONCLUSION

The cleft type did not influence the adaptation of feeding plates manufactured by the conventional method. On the other hand, the adaptation of 3D‐printed plates varied according to the cleft type.

The adaptation of feeding plates manufactured with both techniques was lower in the right palate region regardless of the cleft type. This may be because of an undercut on the initial model, considering that all main models were obtained from the same plaster model. Location‐based differences in feeding plate adaptation should be further investigated in future studies. Feeding plates manufactured with a 3D printer showed higher adaptation than plates manufactured with a conventional method, regardless of measurement region and cleft type. Therefore, considering the higher adaptation, 3D printing can be recommended as the manufacturing method rather than the conventional method for feeding plates.

## CONFLICT OF INTEREST STATEMENT

The authors deny any conflicts of interest in regard to the current study.
